# 3-(3-Fluoro­phenyl­sulfin­yl)-2,4,6,7-tetra­methyl-1-benzofuran

**DOI:** 10.1107/S1600536811016102

**Published:** 2011-05-07

**Authors:** Hong Dae Choi, Pil Ja Seo, Byeng Wha Son, Uk Lee

**Affiliations:** a Department of Chemistry, Dongeui University, San 24 Kaya-dong Busanjin-gu, Busan 614-714, Republic of Korea; b Department of Chemistry, Pukyong National University, 599-1 Daeyeon 3-dong, Nam-gu, Busan 608-737, Republic of Korea

## Abstract

In the title compound, C_18_H_17_FO_2_S, the 3-fluoro­phenyl ring makes a dihedral angle of 78.30 (5)° with the mean plane of the benzofuran fragment. In the crystal, mol­ecules are linked by weak inter­molecular C—H⋯O hydrogen bonds and C—H⋯π inter­actions.

## Related literature

For the biological activity of benzofuran compounds, see: Aslam *et al.* (2009 ▶); Galal *et al.* (2009 ▶); Khan *et al.* (2005 ▶). For natural products with benzofuran rings, see: Akgul & Anil (2003 ▶); Soekamto *et al.* (2003 ▶). For structural studies of related 3-(4-fluoro­phenyl­sulfin­yl)-2-methyl-1-benzofuran derivatives, see: Choi *et al.* (2010**a* ▶,b* ▶).
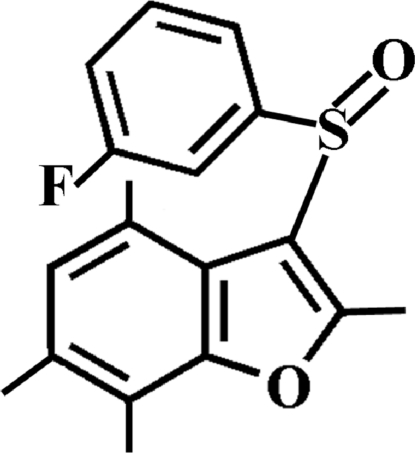


## Experimental

### 

#### Crystal data

C_18_H_17_FO_2_S
*M* *_r_* = 316.38
Triclinic, 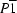

*a* = 7.2690 (2) Å
*b* = 8.0039 (3) Å
*c* = 13.7968 (5) Å
α = 76.839 (1)°
β = 88.561 (1)°
γ = 77.506 (1)°
*V* = 762.86 (4) Å^3^
*Z* = 2
Mo *K*α radiation
μ = 0.23 mm^−1^
*T* = 173 K
0.29 × 0.24 × 0.09 mm

#### Data collection

Bruker SMART APEXII CCD diffractometer
Absorption correction: multi-scan (*SADABS*; Bruker, 2009 ▶) *T*_min_ = 0.936, *T*_max_ = 0.979
13783 measured reflections
3520 independent reflections
2958 reflections with *I* > 2σ(*I*)
*R*_int_ = 0.032

#### Refinement

*R*[*F* ^2^ > 2σ(*F* ^2^)] = 0.041
*wR*(*F* ^2^) = 0.107
*S* = 1.05
3520 reflections
203 parameters
H-atom parameters constrained
Δρ_max_ = 0.35 e Å^−3^
Δρ_min_ = −0.30 e Å^−3^

### 

Data collection: *APEX2* (Bruker, 2009 ▶); cell refinement: *SAINT* (Bruker, 2009 ▶); data reduction: *SAINT*; program(s) used to solve structure: *SHELXS97* (Sheldrick, 2008 ▶); program(s) used to refine structure: *SHELXL97* (Sheldrick, 2008 ▶); molecular graphics: *ORTEP-3* (Farrugia, 1997 ▶) and *DIAMOND* (Brandenburg, 1998 ▶); software used to prepare material for publication: *SHELXL97*.

## Supplementary Material

Crystal structure: contains datablocks global, I. DOI: 10.1107/S1600536811016102/zq2099sup1.cif

Structure factors: contains datablocks I. DOI: 10.1107/S1600536811016102/zq2099Isup2.hkl

Supplementary material file. DOI: 10.1107/S1600536811016102/zq2099Isup3.cml

Additional supplementary materials:  crystallographic information; 3D view; checkCIF report

## Figures and Tables

**Table 1:** Hydrogen-bond geometry (Å, °) *Cg* is the centroid of the C2–C7 benzene ring.

*D*—H⋯*A*	*D*—H	H⋯*A*	*D*⋯*A*	*D*—H⋯*A*
C12—H12*C*⋯O2^i^	0.98	2.34	3.295 (2)	165
C10—H10*A*⋯*Cg*^ii^	0.98	2.74	3.547 (2)	141

Symmetry codes: (i) 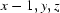; (ii) 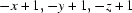.

**Fig. 1.**
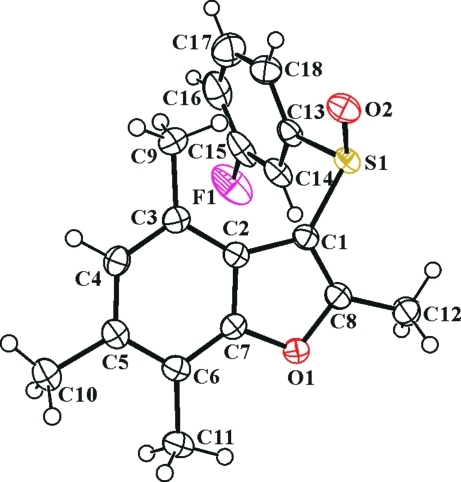
The molecular structure of the title compound with the atom numbering scheme. Displacement ellipsoids are drawn at the 50% probability level., H atoms are presented as a small spheres of arbitrary radius.

**Fig. 2.**
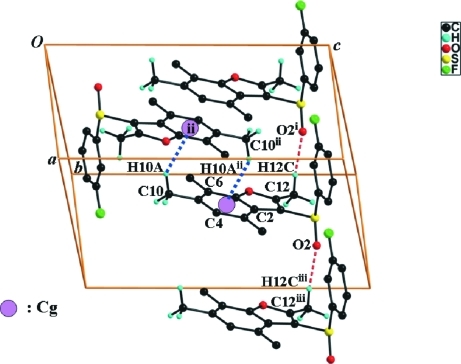
A view of the C—H···O and C—H···π interactions (dotted lines) in the crystal structure of the title compound [symmetry codes: (i) x - 1, y, z; (ii) - x + 1, - y + 1, - z + 1; (iii) x + 1, y, z]

## supplementary crystallographic information

### Comment

A series of benzofuran ring system show interesting pharmacological properties such as antibacterial and antifungal, antitumor and antiviral, and antimicrobial activities (Aslam *et al.*, 2009; Galal *et al.*, 2009; Khan *et al.*, 2005). These compounds occur in a wide range of natural products (Akgul & Anil, 2003; Soekamto *et al.*, 2003). As a part of our study of the substituent effect on the solid state structures of 3-(4-fluorophenylsulfinyl)-2-methyl-1-benzofuran analogues (Choi *et al.*, 2010**a*,b*), we report the crystal structure of the title compound.

In the title compound (Fig. 1), the benzofuran unit is essentially planar, with a mean deviation of 0.016 (1) Å from the least-squares plane defined by the nine constituent atoms. The 3-fluorophenyl ring makes a dihedral angle of 78.30 (5)° with the mean plane of the benzofuran fragment. The crystal packing (Fig. 2) is stabilized by weak intermolecular C—H···O hydrogen bonds between a methyl H atom and the O atom of the sulfinyl group (Table 1; C12—H12C···O2^i^). The crystal structure is further stabilized by intermolecular C—H···π interactions between a methyl H atom and the benzene ring (Table 1; C10—H10A···Cg^ii^, Cg being the centroid of the C2–C7 benzene ring).

### Experimental

77% 3-chloroperoxybenzoic acid (269 mg, 1.2 mmol) was added in small portions to a stirred solution of 3-(3-fluorophenylsulfanyl)-2,4,6,7-tetramethyl-1-benzofuran (360 mg, 1.2 mmol) in dichloromethane (40 mL) at 273 K. After being stirred at room temperature for 5h, the mixture was washed with saturated sodium bicarbonate solution and the organic layer was separated, dried over magnesium sulfate, filtered and concentrated at reduced pressure. The residue was purified by column chromatography (hexane–ethyl acetate, 2:1 v/v) to afford the title compound as a colorless solid [yield 79%, m.p. 413–414 K; *R*_f_ = 0.58 (hexane–ethyl acetate, 2:1 v/v)]. Single crystals suitable for X-ray diffraction were prepared by slow evaporation of a solution of the title compound in acetone at room temperature.

### Refinement

All H atoms were positioned geometrically and refined using a riding model, with C—H = 0.95 Å for aryl and 0.98 Å for methyl H atoms (with *U*_iso_(H) = 1.2*U*_eq_(C) for aryl and 1.5*U*_eq_(C) for methyl H atoms).

### Crystal data

**Table:**

C_18_H_17_FO_2_S	*Z* = 2
*M**_r_* = 316.38	*F*(000) = 332
Triclinic, *P*1	*D*_x_ = 1.377 Mg m^−^^3^
Hall symbol: -P 1	Mo *K*α radiation, λ = 0.71073 Å
*a* = 7.2690 (2) Å	Cell parameters from 4445 reflections
*b* = 8.0039 (3) Å	θ = 2.7–27.1°
*c* = 13.7968 (5) Å	µ = 0.23 mm^−^^1^
α = 76.839 (1)°	*T* = 173 K
β = 88.561 (1)°	Block, colourless
γ = 77.506 (1)°	0.29 × 0.24 × 0.09 mm
*V* = 762.86 (4) Å^3^	

### Data collection

**Table:**

Bruker SMART APEXII CCD diffractometer	3520 independent reflections
Radiation source: rotating anode	2958 reflections with *I* > 2σ(*I*)
graphite multilayer	*R*_int_ = 0.032
Detector resolution: 10.0 pixels mm^-1^	θ_max_ = 27.5°, θ_min_ = 1.5°
φ and ω scans	*h* = −9→9
Absorption correction: multi-scan (*SADABS*; Bruker, 2009)	*k* = −9→10
*T*_min_ = 0.936, *T*_max_ = 0.979	*l* = −17→17
13783 measured reflections	

### Refinement

**Table:**

Refinement on *F*^2^	Primary atom site location: structure-invariant direct methods
Least-squares matrix: full	Secondary atom site location: difference Fourier map
*R*[*F*^2^ > 2σ(*F*^2^)] = 0.041	Hydrogen site location: difference Fourier map
*wR*(*F*^2^) = 0.107	H-atom parameters constrained
*S* = 1.05	*w* = 1/[σ^2^(*F*_o_^2^) + (0.0482*P*)^2^ + 0.326*P*] where *P* = (*F*_o_^2^ + 2*F*_c_^2^)/3
3520 reflections	(Δ/σ)_max_ < 0.001
203 parameters	Δρ_max_ = 0.35 e Å^−^^3^
0 restraints	Δρ_min_ = −0.30 e Å^−^^3^

### Special details

**Table:**

Geometry. All esds (except the esd in the dihedral angle between two l.s. planes) are estimated using the full covariance matrix. The cell esds are taken into account individually in the estimation of esds in distances, angles and torsion angles; correlations between esds in cell parameters are only used when they are defined by crystal symmetry. An approximate (isotropic) treatment of cell esds is used for estimating esds involving l.s. planes.
Refinement. Refinement of F^2^ against ALL reflections. The weighted R-factor wR and goodness of fit S are based on F^2^, conventional R-factors R are based on F, with F set to zero for negative F^2^. The threshold expression of F^2^ > 2sigma(F^2^) is used only for calculating R-factors(gt) etc. and is not relevant to the choice of reflections for refinement. R-factors based on F^2^ are statistically about twice as large as those based on F, and R- factors based on ALL data will be even larger.

### Fractional atomic coordinates and isotropic or equivalent isotropic displacement parameters (Å^2^)

**Table:**

	*x*	*y*	*z*	*U*_iso_*/*U*_eq_	
S1	0.60154 (6)	0.80711 (5)	0.84314 (3)	0.02726 (13)	
F1	0.4058 (2)	0.21849 (18)	0.90810 (10)	0.0677 (4)	
O1	0.24868 (15)	0.91394 (15)	0.61908 (8)	0.0268 (3)	
O2	0.78749 (17)	0.85793 (17)	0.83599 (10)	0.0368 (3)	
C1	0.4973 (2)	0.8378 (2)	0.72511 (11)	0.0242 (3)	
C2	0.5606 (2)	0.7783 (2)	0.63519 (11)	0.0231 (3)	
C3	0.7296 (2)	0.6944 (2)	0.59983 (12)	0.0258 (3)	
C4	0.7203 (2)	0.6631 (2)	0.50521 (12)	0.0280 (3)	
H4	0.8330	0.6070	0.4791	0.034*	
C5	0.5554 (2)	0.7087 (2)	0.44574 (12)	0.0268 (3)	
C6	0.3874 (2)	0.7954 (2)	0.47974 (12)	0.0256 (3)	
C7	0.4001 (2)	0.8278 (2)	0.57324 (12)	0.0240 (3)	
C8	0.3124 (2)	0.9188 (2)	0.71057 (12)	0.0263 (3)	
C9	0.9120 (2)	0.6381 (3)	0.65882 (13)	0.0341 (4)	
H9A	0.9155	0.7190	0.7021	0.051*	
H9B	1.0176	0.6398	0.6132	0.051*	
H9C	0.9222	0.5189	0.6996	0.051*	
C10	0.5592 (3)	0.6614 (2)	0.34618 (13)	0.0337 (4)	
H10A	0.4829	0.5736	0.3482	0.050*	
H10B	0.6894	0.6129	0.3308	0.050*	
H10C	0.5076	0.7668	0.2947	0.050*	
C11	0.2058 (2)	0.8485 (2)	0.42018 (13)	0.0332 (4)	
H11A	0.1065	0.9088	0.4573	0.050*	
H11B	0.1705	0.7437	0.4077	0.050*	
H11C	0.2219	0.9279	0.3565	0.050*	
C12	0.1714 (2)	1.0080 (3)	0.77179 (14)	0.0346 (4)	
H12A	0.1189	1.1283	0.7348	0.052*	
H12B	0.2320	1.0110	0.8338	0.052*	
H12C	0.0700	0.9437	0.7873	0.052*	
C13	0.6508 (2)	0.5703 (2)	0.87613 (12)	0.0290 (4)	
C14	0.5051 (3)	0.4843 (2)	0.87530 (12)	0.0346 (4)	
H14	0.3813	0.5471	0.8532	0.042*	
C15	0.5468 (3)	0.3042 (3)	0.90778 (13)	0.0441 (5)	
C16	0.7232 (4)	0.2093 (3)	0.94023 (15)	0.0532 (6)	
H16	0.7472	0.0848	0.9612	0.064*	
C17	0.8648 (3)	0.2981 (3)	0.94177 (16)	0.0540 (6)	
H17	0.9879	0.2346	0.9646	0.065*	
C18	0.8292 (3)	0.4799 (3)	0.91021 (14)	0.0395 (4)	
H18	0.9269	0.5412	0.9121	0.047*	

### Atomic displacement parameters (Å^2^)

**Table:**

	*U*^11^	*U*^22^	*U*^33^	*U*^12^	*U*^13^	*U*^23^
S1	0.0301 (2)	0.0285 (2)	0.0260 (2)	−0.00976 (16)	−0.00194 (15)	−0.00842 (16)
F1	0.1098 (12)	0.0561 (8)	0.0512 (8)	−0.0541 (8)	−0.0068 (7)	−0.0057 (6)
O1	0.0239 (5)	0.0294 (6)	0.0277 (6)	−0.0050 (4)	−0.0006 (4)	−0.0080 (5)
O2	0.0341 (6)	0.0403 (7)	0.0420 (7)	−0.0173 (6)	−0.0034 (5)	−0.0121 (6)
C1	0.0258 (7)	0.0234 (8)	0.0245 (8)	−0.0078 (6)	−0.0007 (6)	−0.0050 (6)
C2	0.0266 (7)	0.0191 (7)	0.0236 (7)	−0.0075 (6)	−0.0003 (6)	−0.0026 (6)
C3	0.0264 (8)	0.0219 (8)	0.0287 (8)	−0.0064 (6)	−0.0001 (6)	−0.0040 (6)
C4	0.0291 (8)	0.0237 (8)	0.0306 (8)	−0.0050 (6)	0.0039 (6)	−0.0063 (7)
C5	0.0347 (8)	0.0221 (8)	0.0246 (8)	−0.0101 (6)	0.0016 (6)	−0.0038 (6)
C6	0.0299 (8)	0.0215 (8)	0.0251 (8)	−0.0082 (6)	−0.0027 (6)	−0.0016 (6)
C7	0.0243 (7)	0.0204 (7)	0.0264 (8)	−0.0057 (6)	0.0013 (6)	−0.0031 (6)
C8	0.0276 (8)	0.0262 (8)	0.0273 (8)	−0.0101 (6)	0.0005 (6)	−0.0062 (6)
C9	0.0256 (8)	0.0397 (10)	0.0354 (9)	−0.0007 (7)	−0.0017 (7)	−0.0112 (8)
C10	0.0421 (10)	0.0331 (10)	0.0283 (9)	−0.0109 (8)	0.0018 (7)	−0.0097 (7)
C11	0.0341 (9)	0.0362 (10)	0.0292 (9)	−0.0081 (7)	−0.0062 (7)	−0.0058 (7)
C12	0.0271 (8)	0.0435 (11)	0.0365 (9)	−0.0073 (7)	0.0025 (7)	−0.0162 (8)
C13	0.0397 (9)	0.0287 (9)	0.0195 (7)	−0.0090 (7)	−0.0013 (6)	−0.0055 (6)
C14	0.0468 (10)	0.0362 (10)	0.0243 (8)	−0.0163 (8)	−0.0020 (7)	−0.0069 (7)
C15	0.0786 (15)	0.0390 (11)	0.0243 (9)	−0.0312 (10)	0.0018 (9)	−0.0094 (8)
C16	0.0924 (18)	0.0281 (10)	0.0355 (11)	−0.0065 (11)	−0.0036 (11)	−0.0056 (8)
C17	0.0620 (14)	0.0423 (12)	0.0468 (12)	0.0052 (10)	−0.0067 (10)	−0.0028 (10)
C18	0.0415 (10)	0.0403 (11)	0.0340 (10)	−0.0055 (8)	−0.0040 (8)	−0.0053 (8)

### Geometric parameters (Å, °)

**Table:**

S1—O2	1.4883 (12)	C9—H9C	0.9800
S1—C1	1.7565 (16)	C10—H10A	0.9800
S1—C13	1.8024 (18)	C10—H10B	0.9800
F1—C15	1.351 (2)	C10—H10C	0.9800
O1—C8	1.3670 (19)	C11—H11A	0.9800
O1—C7	1.3862 (18)	C11—H11B	0.9800
C1—C8	1.358 (2)	C11—H11C	0.9800
C1—C2	1.457 (2)	C12—H12A	0.9800
C2—C7	1.395 (2)	C12—H12B	0.9800
C2—C3	1.400 (2)	C12—H12C	0.9800
C3—C4	1.390 (2)	C13—C18	1.377 (3)
C3—C9	1.501 (2)	C13—C14	1.384 (2)
C4—C5	1.402 (2)	C14—C15	1.377 (3)
C4—H4	0.9500	C14—H14	0.9500
C5—C6	1.395 (2)	C15—C16	1.369 (3)
C5—C10	1.504 (2)	C16—C17	1.374 (3)
C6—C7	1.382 (2)	C16—H16	0.9500
C6—C11	1.500 (2)	C17—C18	1.389 (3)
C8—C12	1.479 (2)	C17—H17	0.9500
C9—H9A	0.9800	C18—H18	0.9500
C9—H9B	0.9800		
			
O2—S1—C1	111.73 (7)	C5—C10—H10B	109.5
O2—S1—C13	106.34 (8)	H10A—C10—H10B	109.5
C1—S1—C13	98.05 (7)	C5—C10—H10C	109.5
C8—O1—C7	106.92 (12)	H10A—C10—H10C	109.5
C8—C1—C2	107.56 (14)	H10B—C10—H10C	109.5
C8—C1—S1	118.37 (12)	C6—C11—H11A	109.5
C2—C1—S1	133.75 (12)	C6—C11—H11B	109.5
C7—C2—C3	118.54 (14)	H11A—C11—H11B	109.5
C7—C2—C1	104.30 (13)	C6—C11—H11C	109.5
C3—C2—C1	137.14 (14)	H11A—C11—H11C	109.5
C4—C3—C2	116.01 (14)	H11B—C11—H11C	109.5
C4—C3—C9	120.60 (15)	C8—C12—H12A	109.5
C2—C3—C9	123.38 (15)	C8—C12—H12B	109.5
C3—C4—C5	124.32 (15)	H12A—C12—H12B	109.5
C3—C4—H4	117.8	C8—C12—H12C	109.5
C5—C4—H4	117.8	H12A—C12—H12C	109.5
C6—C5—C4	119.95 (15)	H12B—C12—H12C	109.5
C6—C5—C10	120.06 (15)	C18—C13—C14	121.39 (17)
C4—C5—C10	119.98 (15)	C18—C13—S1	119.02 (14)
C7—C6—C5	114.88 (14)	C14—C13—S1	119.32 (14)
C7—C6—C11	122.22 (15)	C15—C14—C13	117.24 (18)
C5—C6—C11	122.90 (15)	C15—C14—H14	121.4
C6—C7—O1	123.28 (14)	C13—C14—H14	121.4
C6—C7—C2	126.23 (15)	F1—C15—C16	118.97 (19)
O1—C7—C2	110.48 (13)	F1—C15—C14	117.9 (2)
C1—C8—O1	110.73 (14)	C16—C15—C14	123.1 (2)
C1—C8—C12	133.72 (15)	C15—C16—C17	118.52 (19)
O1—C8—C12	115.56 (14)	C15—C16—H16	120.7
C3—C9—H9A	109.5	C17—C16—H16	120.7
C3—C9—H9B	109.5	C16—C17—C18	120.5 (2)
H9A—C9—H9B	109.5	C16—C17—H17	119.8
C3—C9—H9C	109.5	C18—C17—H17	119.8
H9A—C9—H9C	109.5	C13—C18—C17	119.2 (2)
H9B—C9—H9C	109.5	C13—C18—H18	120.4
C5—C10—H10A	109.5	C17—C18—H18	120.4
			
O2—S1—C1—C8	−133.77 (13)	C8—O1—C7—C2	−0.24 (17)
C13—S1—C1—C8	115.00 (14)	C3—C2—C7—C6	3.1 (2)
O2—S1—C1—C2	53.79 (18)	C1—C2—C7—C6	−178.02 (15)
C13—S1—C1—C2	−57.45 (17)	C3—C2—C7—O1	−177.96 (13)
C8—C1—C2—C7	−1.30 (17)	C1—C2—C7—O1	0.94 (17)
S1—C1—C2—C7	171.73 (13)	C2—C1—C8—O1	1.22 (18)
C8—C1—C2—C3	177.28 (18)	S1—C1—C8—O1	−173.06 (10)
S1—C1—C2—C3	−9.7 (3)	C2—C1—C8—C12	−178.76 (18)
C7—C2—C3—C4	−1.8 (2)	S1—C1—C8—C12	7.0 (3)
C1—C2—C3—C4	179.78 (17)	C7—O1—C8—C1	−0.64 (17)
C7—C2—C3—C9	178.81 (15)	C7—O1—C8—C12	179.35 (14)
C1—C2—C3—C9	0.4 (3)	O2—S1—C13—C18	14.28 (16)
C2—C3—C4—C5	−0.4 (2)	C1—S1—C13—C18	129.83 (15)
C9—C3—C4—C5	179.01 (16)	O2—S1—C13—C14	−171.55 (13)
C3—C4—C5—C6	1.7 (3)	C1—S1—C13—C14	−56.01 (14)
C3—C4—C5—C10	−177.23 (15)	C18—C13—C14—C15	−1.5 (3)
C4—C5—C6—C7	−0.5 (2)	S1—C13—C14—C15	−175.51 (13)
C10—C5—C6—C7	178.34 (14)	C13—C14—C15—F1	179.53 (15)
C4—C5—C6—C11	179.90 (15)	C13—C14—C15—C16	0.1 (3)
C10—C5—C6—C11	−1.2 (2)	F1—C15—C16—C17	−178.53 (18)
C5—C6—C7—O1	179.35 (14)	C14—C15—C16—C17	0.9 (3)
C11—C6—C7—O1	−1.1 (2)	C15—C16—C17—C18	−0.5 (3)
C5—C6—C7—C2	−1.8 (2)	C14—C13—C18—C17	1.8 (3)
C11—C6—C7—C2	177.74 (15)	S1—C13—C18—C17	175.89 (15)
C8—O1—C7—C6	178.75 (14)	C16—C17—C18—C13	−0.8 (3)

### Hydrogen-bond geometry (Å, °)

**Table:**

Cg is the centroid ofthe C2–C7 benzene ring.

**Table:**

*D*—H···*A*	*D*—H	H···*A*	*D*···*A*	*D*—H···*A*
C12—H12C···O2^i^	0.98	2.34	3.295 (2)	165.
C10—H10A···Cg^ii^	0.98	2.74	3.547 (2)	141.

Symmetry codes: (i) *x*−1, *y*, *z*; (ii) −*x*+1, −*y*+1, −*z*+1.
